# Low-Temperature Synthesis and Characterization of Hybrid Heavy Metal Zero- Dimensional Cluster Compounds Containing Pt/Ir and Bi.

**DOI:** 10.1063/4.0001111

**Published:** 2025-10-27

**Authors:** Gayomi Kanchana Samarakoon Mudiyanselage, Logan Schilling, Sviatoslav Baranets

**Affiliations:** Louisiana State Univesity, Baton Rouge, Louisiana, USA

**Keywords:** Cluster, Ionic Liquids, Unit Cell Parameters, DFT, HOMO–LUMO Gap

## Abstract

Three novel hybrid organic-inorganic cluster compounds comprising 5*d* heavy metals (*M* = Pt, Ir) and Bi were synthesized at 180 °C in ionic liquid (IL) 1-butyl-3-methylimidazolium tetrafluoroborate ([BMIm][BF_4_]), which served both as reactant and reaction medium. Stoichiometric reactions between the appropriate metal halide and [BMIm][BF_4_] yielded three distinct cluster-containing salts: [PtBi_6_Cl_12_][BMIm]_2_, [Pt_2_Bi_12_Br_22_][BMIm]_2_, and [IrBi_6_Cl_12_][BMIm]_3_, all crystallizing in three different structure types. [PtBi_6_Cl_12_][BMIm]_2_ crystallizes in the monoclinic space group *P*2_1_/*c* with the unit cell parameters of *a* = 9.1154(5), *b* = 9.0692(4), *c* = 23.8159(13), and *β* = 93.890(2) while [IrBi_6_Cl_12_][BMIm]_3_ crystallizes in the monoclinic space group *C*2/*c* (*a* = 22.0181(7), *b* = 12.6726(4), *c* = 35.2412(11), *β* = 95.132(1)) both featuring cuboctahedral [*M*Bi_6_Cl_12_]*^x^*^–^ (*x* = 2, 3) anionic clusters. The Br-bearing analog [Pt_2_Bi_12_Br_22_][BMIm]_2_ crystallizes in the triclinic space group *P* (*a* = 9.3063(3), *b* = 12.4615(4), *c* = 14.7424(5), *α* =70.484 (10), *β* = 87.966(10), γ = 87.497(10)) and exhibits double cuboctahedra [PtBi_12_Br_22_]^2–^ cluster. Each cluster anion is charge-balanced by [BMIm]^+^ ions, providing a unique combination of the lightest and heaviest elements in the title compounds. Molecular DFT calculations reveal that each anionic cluster, [PtBi_6_Cl_12_]^2–^, [IrBi_6_Cl_12_]^3–^, [PtBi_12_Br_22_]^2–^ has HOMO–LUMO gaps of 3.3774 eV, 3.2332 eV and 2.4106 eV respectively. Details of the synthesis, crystal and electronic structures, and perspectives for semiconducting applications are discussed.

